# Associations of left renal vein entrapment with IgA nephropathy and Henoch–Schönlein purpura nephritis

**DOI:** 10.1080/0886022X.2022.2118065

**Published:** 2022-09-07

**Authors:** Fengmei Wang, Huizi Zhu, Shougang Bao, Hengtao Qi, Liang Xu, Xiang Liu, Chunjuan Zhai, Xiaowei Yang, Rong Wang

**Affiliations:** a Institute of Nephrology, Zhong Da Hospital, Southeast University School of Medicine, Nanjing, P. R. China; b Department of Nephrology, Shandong Provincial Hospital, Shandong University, Jinan, P. R. China; c Department of Ultrasound, Shandong Provincial Hospital Affiliated to Shandong First Medical University, Jinan, P. R. China; d Department of Nephrology, Shandong Provincial Hospital Affiliated to Shandong First Medical University, Jinan, P. R. China; e Department of Cardiology, Shandong Provincial Hospital affiliated to Shandong First Medical University, Jinan, P. R. China

**Keywords:** Left renal vein entrapment, biopsy-proven renal diseases, IgA nephropathy, Henoch–Schönlein purpura nephritis

## Abstract

**Objectives:**

The aims of the study were to identify whether left renal vein (LRV) entrapment was more prevalent in IgA nephropathy (IgAN) and Henoch–Schönlein purpura nephritis (HSPN) compared with other types of renal diseases, and the association of LRV entrapment with glomerular incidental IgA and galactose-deficient-IgA1 (Gd-IgA1) deposition.

**Methods:**

A total of 797 patients with biopsy-proven kidney diseases have been screened for LRV entrapment by color Doppler ultrasound, and the prevalence of LRV entrapment in different types of renal diseases were then analyzed. Propensity score matching analysis was used to adjust for age, gender, and body mass index. Immunostaining of Gd-IgA1 with KM55 was performed in paraffin-embedded sections of renal biopsy specimens.

**Results:**

LRV entrapment was diagnosed in 47 patients (6%) with several kinds of renal diseases in our cohort. A total of 32 (68%) LRV entrapments were combined with expanded IgAN (idiopathic IgAN and HSPN). The prevalence of LRV entrapment in expanded IgAN was significantly higher than that in non-expanded IgAN (17 vs. 2%, *p* < 0.001), even after adjustment for age, gender, and body mass index by propensity score matching analysis (13 vs. 2%, *p* < 0.001). Removing expanded IgAN and LN, glomerular incidental IgA deposition was observed to be significantly more common in patients with LRV entrapment compared with patients without it (43 vs. 9%, *p* < 0.001). Furthermore, in glomerular diseases with incidental IgA deposits, significantly much larger proportion of patients with LRV entrapment were positive for glomerular Gd-IgA1 in contrast to patients without LRV entrapment (5/5 *vs.* 5/17, *p* = 0.01).

**Conclusions:**

LRV entrapment coexisted with several kinds of renal diseases, with a significantly higher prevalence in patients with idiopathic IgAN and HSPN. In patients of LN and IgAN-unrelated disease with LRV entrapment, glomerular IgA and Gd-IgA1 deposition was more common compared with patients without LRV entrapment.

## Introduction

Left renal vein (LRV) entrapment, also known as nutcracker phenomenon (NCP), refers to the entrapment of LRV most commonly between abdominal aorta and superior mesenteric artery (SMA) [1]. NCP sometimes accompanies various clinical symptoms, which is then termed nutcracker syndrome (NCS).

NCS could be differentiated clinically into two subtypes as follows: renal presentations and non-renal presentations [2]. Hematuria, orthostatic proteinuria with or without flank pain is the common renal clinical presentations [3]. The non-renal presentations include abdominal pain, varicocele, dyspareunia, dysmenorrhea, fatigue, and orthostatic intolerance and so on [4]. NCP-related hematuria is generally considered to be caused by elevated LRV pressure resulting in the rupture of thin-walled septum between the varices and the collecting system in the renal fornix. The mechanisms of NCP-related proteinuria have not been well clarified yet, one suggested reason is the elevation of levels of norepinephrine and angiotensin II due to the changes of renal hemodynamics [5].

LRV entrapment has been described to coexist with idiopathic glomerular diseases in some case reports [6–10]. Among these cases, coexistence with IgA nephropathy (IgAN) and Henoch–Schönlein purpura nephritis (HSPN) were the most commonly reported ones. In an analysis of renal pathological types in Chinese isolated hematuria patients combined with glomerular nephritis and NCP, 69% (20/29) of the patients were diagnosed with IgAN [11]. Imai Naohiko et al. have shown that the prevalence of LRV entrapment in IgAN was 6.8% (10/146) in Japanese patients [12]. Recently, we have reported a rare case of concurrent nutcracker and SMA syndrome in a 15-year-old Chinese male juvenile diagnosed with IgAN [13]. Considering the relatively common combination of LRV entrapment and IgAN, a possible causal relationship between them has been raised [6,12]. However, the exact population prevalence of NCP is unknown, and there is no available data to determine whether NCP is also prevalent in other types of renal diseases at present.

In this study, we screened LRV entrapment in a large cohort of Chinese patients with biopsy-proven renal diseases, and explored the association of LRV entrapment with the presence of IgAN and glomerular incidental IgA deposition.

## Patients and methods

### Patients

We prospectively screened all patients admitted to Shandong Provincial Hospital affiliated to Shandong First Medical University with biopsy-proven kidney diseases for LRV entrapment by Doppler ultrasound from 2 January 2019 to 31 August 2021. Written informed consent was obtained for renal biopsy and screening of LRV entrapment from each patient. For participants under 16 years old, written informed consent was provided by a parent or guardian. The research was in compliance of the Declaration of Helsinki. The study was approved by the local ethics committees of Shandong Provincial Hospital (the approval number was NO. 2019-072).

### Clinical evaluation and renal histopathology

The following clinical data were collected and analyzed: age, gender, height, weight, serum albumin, serum creatinine (Scr), 24-h urine protein, hematuria, and estimated GFR (eGFR). eGFR was calculated using a Scr-based equation adjusted for coefficients for age and gender by modified abbreviated MDRD equation based on data from Chinese CKD patients: eGFR (ml/min per 1.73 m^2^) =175 × [Scr (mg/dL)]^−1.234^ × age^−0.179^ × (0.79 if female) [14]. For adolescents, eGFR calculated by using the Schwartz equation. According to the Schwartz equation, we used the following formula to calculate eGFR: k × patient’s length (cm)/Scr (mg/dL). The value of k was 0.55 for adolescent girls, while it was 0.7 for adolescent males. We normalized the eGFR to the ideal body weight‐derived body surface area [15].

In the local clinical practice, the criteria of renal biopsy were usually as follows: proteinuria >1 g/24 h with or without hematuria; proteinuria >0.5 g/24 h with hematuria; renal insufficiency. The renal biopsy specimens were examined by light microscopy, direct immunofluorescence, and electron microscopy.

### Double immunofluorescent stain of Gd-IgA1 and IgA

Glomerular Gd-IgA1 and IgA deposition were examined by double immunofluorescent staining as described [16]. Briefly, paraffin-embedded sections of 3 μm thickness were prepared for staining. After deparaffinization with a series of xylene and ethanol concentrations and subsequent rehydration, antigen retrieval using 0.05% bacterial protease subtilisin A (Sigma-Aldrich, Tokyo, Japan) dissolved in 5 mmol/L Tris (hydroxymethyl)-aminomethane buffer (pH 7.6) was performed at room temperature for 2 h. Samples were then rinsed with phosphate-buffered saline (PBS) and blocked with 5% bovine serum albumin (BSA) at room temperature for 30 min. Sections were incubated for 1 h at 37 °C with rat monoclonal anti-human Gd-IgA1 antibody (KM55) (100 mg/mL, Immuno-Biological Laboratories, Fujioka-Shi, Japan). After several washes with triethanolamine-buffered saline containing 0.05% tween-20 (TBST), Alexa Fluor 555-conjugated goat anti-rat IgG antibody (1:1000, Abcam, Cambridge, UK) was incubated with the samples at 37 °C for 30 min. Samples were washed with TBST and incubated with FITC–conjugated polyclonal rabbit anti-human IgA antibody (1:40; ZSGB-BIO, Beijing, China) at 37 °C for 30 min. After washing with TBST, slides were sealed with Fluoromount (Solarbio, Beijing, China). For microscopic observation of immunostained samples, a fluorescence microscope (OLYMPUS BX63, Tokyo, Japan), and a confocal microscope (Leica DM6, Wetzlar, Germany) were used.

### Diagnosis of renal diseases

Renal disease diagnosis was made by comprehensive judgment of clinical and pathological manifestations. All the patients diagnosed with idiopathic IgAN in our study had been examined carefully to exclude liver disease and bowel disease which are suggested to be common causes of secondary IgAN [17]. The diagnosis of HSPN was confirmed by clinical manifestations and renal pathological changes, and the updated Oxford classification of IgAN was used for patients with HSPN [18].

### Diagnosis of nutcracker phenomenon by color Doppler sonography

Compression of LRV between abdominal aorta and SMA was defined as anterior LRV entrapment, and compression of LRV between abdominal aorta and vertebral column was categorized as posterior LRV entrapment. The main standards for ultrasound diagnosis of NCP are described as follows, according to previous studies with mild modification [19–21]: (1) the angle between SMA and abdominal aorta is less than 30°; (2) the flow velocity of stenosis of the LRV in the supine position accelerates remarkably with a ratio higher than 5 between the Doppler ultrasound peak velocity of the narrow tract and the distended portion; (3) the inner diameter ratio between ratio between the renal hilum and stenosis of the LRV in the supine position is >3; (4) the LRV entrapment with collateral circulation in the left lumbar ascending vein. LRV entrapment was screened by 2 independent sonologists with 10-year experience in vessel ultrasound without knowledge of the biopsy results, and comprehensive judgment was given according to the diagnostic criteria. When discrepancy occurred, agreement was reached after discussion.

### Statistical analysis

Statistical software SPSS version 25.0 (IBM SPSS Statistics for Windows, IBM Corp., Armonk, NY, USA) was employed for all the statistical analysis. Quantitative data were expressed as mean ± s.d., median with range (minimum and maximum), or number (%). For comparison of clinical features of patients, *t*-tests, the Mann–Whitney *U*-tests, and *χ*^2^ test were used. Propensity score matching (PSM) analysis was used to compare the prevalence of LRV entrapment in patients with and without expanded IgAN. Nearest neighbor 1:2 PSM for the following 3 variables was performed: age, sex, and BMI. Statistical significance was considered as *p* < 0.05.

## Results

### General data of patients with biopsy-proven renal diseases

In total, 797 patients with biopsy-proven kidney diseases have been screened for LRV entrapment over the study period. The median age of the patients was 44 years and ranged from 12 to 81 years. The male-to-female ratio was 1.32:1. The median BMI was 25.1 kg/m^2^, and ranged from 13.9 to 47.8 kg/m^2^.

Glomerular diseases comprised 98% of the total biopsied cases. The most common idiopathic glomerulonephritis was primary membranous nephropathy (PMN) (43%), which was followed by IgAN at 19%. There were 35 (4%) patients with HSPN enrolled in our study (Table 1).

**Table 1.** The general data of patients with biopsy-proven renal diseases.

Characteristic	Value
Demographic data	
Gender (male/female)	453/344
Age (median, range) (years)	44 (12, 81)
BMI (median, range) (kg/m^2^)	25.1 (13.9–47.8)
Idiopathic glomerular disease	No. (%)
IgAN	152 (19%)
PMN	340 (43%)
FSGS	37 (5%)
MCD	76 (10%)
C3 glomerular nephritis	1 (0.1%)
Secondary glomerular disease	No. (%)
HSPN	35 (4%)
Diabetic kidney disease	34 (4%)
LN	50 (6%)
Renal amyloidosis	7 (0.9%)
HBV-glomerular nephritis	3 (0.4%)
Others	43 (5%)
Renal tubulointerstitial disease	19 (2%)

BMI: body mass index; IgAN: IgA nephropathy; PMN: primary membranous nephropathy; FSGS: focal segmental glomerular sclerosis; MCD: minimal change disease; HSPN: Henoch–Schonlein purpura nephritis; LN: lupus nephritis; HBV: Hepatitis B virus

### LRV entrapment in patients with biopsy-proven renal diseases

LRV entrapment was diagnosed in 47 patients (6%), with 46 anterior types and only 1 posterior type. LRV entrapment was observed to coexist with several kinds of renal diseases, including 10 cases diagnosed with HSPN, 22 cases with IgAN, 10 cases with PMN, 1 case with LN, 1 case with Alport syndrome, 1 case with C3 glomerulonephritis, 1 case with renal tubular interstitial nephritis (TIN) and 1 case with renal amyloidosis. Of the 47 patients, the average age was 32.7 years and ranged from 15 to 67 years, and 33 (70%) were females. The average BMI was 20.5 ± 2.9 kg/m^2^, which was significantly lower than that of patients without LRV entrapment (20.5 ± 2.9 vs. 25.8 ± 4.1, *p* < 0.001).

### The prevalence of LRV entrapment in different types of renal diseases

As shown in Table 2, the prevalence of LRV entrapment in patients with PMN, which was the most common type of renal disease in our cohort, was 3%. The incidence of LRV entrapment in IgAN and HSPN was 14% (22/152) and 29% (10/35), respectively, either of which was significantly higher than that of in PMN (*p* < 0.001, *p* < 0.001, respectively). In patients with other types of renal diseases, the frequency of LRV entrapment was similar to that of in PMN (2 vs. 3%, *p* = 0.406). Patients with IgAN and HSPN were younger and with lower BMI compared with patients with other kidney diseases with statistical significance (Table 2). To identify whether the difference of the distribution of LRV entrapment was due to the discrepancies in age, gender, and shape, we performed the PSM analysis.

**Table 2.** The prevalence of LRV entrapment in different types of renal diseases.

	LRV entrapment%	Age (mean ± s.d.)	Female No. (%)	BMI (mean ± s.d.)
PMN	3 (10/340)	47.6 ± 12.9	129 (38%)	26.0 ± 4.2
IgAN	14 (22/152)*	39.0 ± 13.7*	70 (46%)*	25.0 ± 3.9*
HSPN	29 (10/35)*	35.0 ± 18.2*	18 (51%)	24.1 ± 4.3*
Other types	2 (4/220)	43.8 ± 14.8*	87 (40%)	25.8 ± 4.4

LRV: left renal vein; BMI: body mass index; PMN: primary membranous nephropathy; IgAN: IgA nephropathy; HSPN: Henoch–Schonlein purpura nephritis

*p < 0.05 compared with the related parameters of PMN.

Since HSPN is considered to be a systemic form of IgAN [22,23], we merged patients with IgAN and HSPN into one group (expanded IgAN group). The prevalence of LRV entrapment in expanded IgAN was still significantly higher than that in non-expanded IgAN (17 vs. 2%, *p* < 0.001). The 1:2 PSM yielded matched pairs of 161 patients with expanded IgAN and 322 patients with non-expanded IgAN, resulting in no differences in age, gender, BMI. In the matched cohort, 21 patients (13%) with expanded IgAN were combined with LRV entrapment compared with 8 patients (2%) with non-expanded IgAN (*p* < 0.001) (detailed in Table 3).

**Table 3.** Characteristics of the patients before and after propensity score matching.

	Before matching			After matching		
	Expanded IgAN (*n* = 187)	Non-expanded IgAN (*n* = 610)	*p* Value	Expanded IgAN (*n* = 161)	Non-expanded IgAN (*n* = 322)	*p* Value
Female gender%	47 (88/187)	42 (256/354)	0.238	48 (78/161)	43 (140/322)	0.332
Age (years)	38.3 ± 14.6	44.9 ± 14.2	<0.001	41.6 ±13.1	41.5 ± 13.2	0.965
BMI (kg/m^2^)	24.7 ± 4.0	25.7 ± 4.2	0.011	24.9 ± 3.6	25.5 ± 4.1	0.128
LRV entrapment%	17 (32/187)	2 (15/610)	<0.001	13 (21/161)	2 (8/322)	<0.001

IgAN: IgA nephropathy; BMI: body mass index; LRV: left renal vein

### Clinicopathological features of patients with and without LRV entrapment in IgAN and HSPN

The detailed data of clinicopathological features of patients with and without LRV entrapment in IgAN and HSPN were listed in Supplementary Table 1. The values of BMI in IgAN or HSPN patients with LRV entrapment were significantly much lower than those of patients without LRV entrapment (20.2 ± 3.0 vs. 25.3 ± 3.8 kg/m^2^, *p* < 0.05; 19.3 ± 1.5 vs. 26.1 ± 3.3 kg/m^2^, *p* < 0.05, respectively). In HSPN patients, patients with LRV entrapment had lower levels of baseline 24-h urine protein and higher levels of serum albumin compared with patients without LRV entrapment (0.8 ± 0.4 vs. 1.7 ± 1.1 g/d, p < 0.05; 42.5 ± 4.2 vs. 37.6 ± 5.4 g/l, *p* < 0.05). No obvious difference was found in other clinical and pathological indices.

### Association of LRV entrapment with glomerular incidental IgA and Gd-IgA1 deposition

Since LRV entrapment was more common in expanded IgAN, we further explored the association of LRV entrapment with glomerular incidental IgA deposition in non-expanded IgAN patients. LN is a disease with ‘full-house’ immunofluorescent staining, and only one LRV entrapment was detected in patients with LN in our cohort, we excluded patients with LN in the analysis either. Removing patients with expanded IgAN and LN, there were 560 patients left in the analytic cohort. Among the 560 patients, 53 (9%) had glomerular incidental IgA deposition, and 14 (3%) had LRV entrapment. As shown in Table 4, in LRV entrapment group, 43% (6/14) of patients had glomerular incidental IgA deposits. While in non-LRV entrapment group, glomerular IgA deposits were observed in only 9% (47/546) of patients, the prevalence of which was significantly lower than that of patients with LRV entrapment (*p* < 0.001).

**Table 4.** Association of LRV entrapment with glomerular IgA deposition in patients without expanded-IgAN and LN.

	Glomerular IgA deposits		
	LRV entrapment group (*n* = 14)	Non-LRV entrapment group (*n* = 546)	*p* Value
Total cases	6 (6/14, 43%)	47 (47/546, 9%)	<0.001
PMN	4 (4/10, 60%)	31 (31/330, 9%)	0.020
TIN	1 (1/1,100%)	0 (0/14, 0%)	–
C3 GN	1 (1/1,100%)	0 (0/0, 0%)	–
Other types	0 (0/2, 0%)	16 (16/198, 8%)	–

LRV: left renal vein; IgAN: IgA nephropathy; LN: lupus nephritis; PMN: primary membranous nephropathy; TIN: tubular interstitial nephritis; C3GN: C3 glomerulonephritis

**Figure 1.**
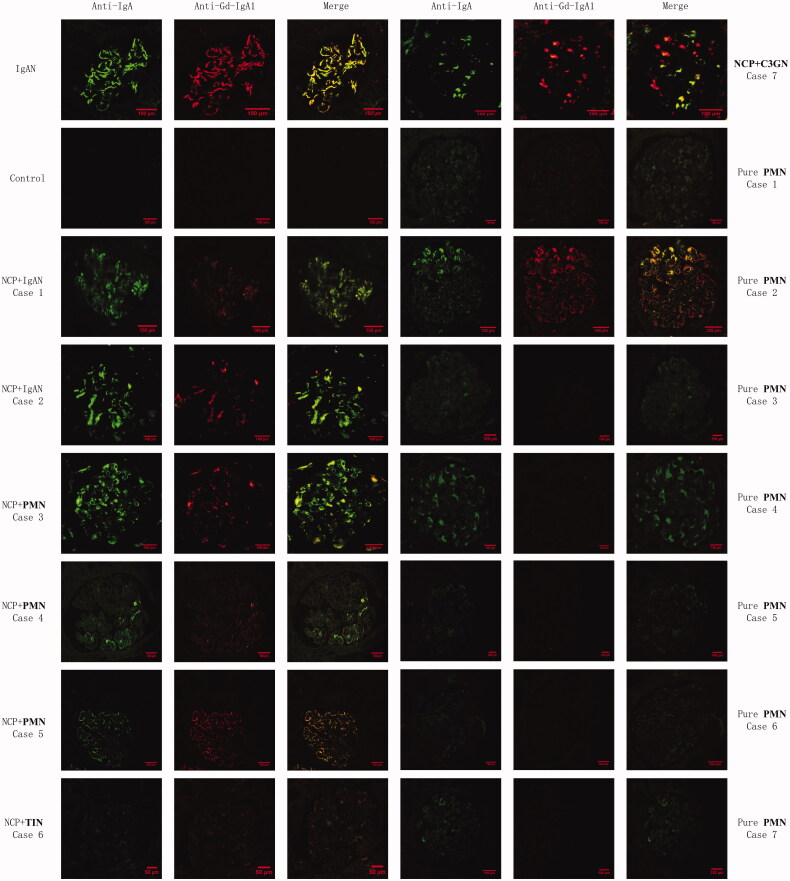
Glomerular deposition of IgA and galactose-deficient IgA1 in patients with different kidney diseases with or without NCP. Double-immunofluorescence staining for IgA and Gd-IgA1. First column, IgA staining; second column, Gd-IgA1 monoclonal antibody (KM55) staining; third column, merged images (Bars = 100μm; original magnification ×400). In patients with IgAN, and IgAN + NCP, glomerular Gd-IgA1 deposition was detected, localized in the mesangial region with IgA. In patients with TIN + NCP, and C3GN + NCP, both Gd-IgA1 and IgA findings were positive, localized in the mesangial region. In patients with PMN + NCP, both Gd-IgA1 and IgA findings were positive along the capillary wall. Meanwhile, in 5 out of 17 patients with pure PMN (without NCP), both Gd-IgA1 and IgA findings were positive along the capillary wall (images were shown as pure PMN Cases 1 and 2). Twelve patients with pure PMN showed only IgA-positive along the capillary wall and Gd-IgA1 negative findings (representative images were shown as pure PMN case 3–7).*Abbreviations:* Gd-IgA1: galactose-deficient-IgA1; IgAN: IgA nephropathy; NCP: nutcracker phenomenon; TIN: tubular interstitial nephritis; C3GN: C3 glomerulonephritis; PMN: primary membranous nephropathy.

Gd-IgA1 has been identified as among the essential effector molecules in the pathogenesis of IgAN. Subsequently, we conducted double-immunofluorescence staining of IgA and Gd-IgA1 in patients with glomerular incidental IgA deposition. As shown in Table 4, in LRV entrapment group, 6 patients had incidental IgA deposits, we got kidney specimens from 5 out of the 6 patients (3 from PMN, 1 from TIN, and 1 from C3 glomerulonephritis), and Gd-IgA1 deposition was observed in all of the 5 patients (Figure 1). In non-LRV entrapment group, 47 patients had incidental IgA deposits. In order to match the pathological types with LRV entrapment patients, we randomly chose 17 PMN patients accompanied by IgA deposition to perform KM55 staining, and Gd-IgA1 was only detected in 5 of the 17 patients (Figure 1). Patients with LRV entrapment were significantly more common to have glomerular incidental Gd-IgA1 deposition (5/5 vs. 5/17, *p* = 0.01).

## Discussion

This study provides the first results of the prevalence of LRV entrapment in different types of biopsy-proven renal diseases. In this large cohort, coexistence of LRV entrapment was significantly more common in expanded IgAN compared with other kidney disease, even after adjusting for age, gender, and BMI. In patients with LN and IgAN-unrelated diseases, LRV entrapment correlated with the frequency of glomerular incidental IgA and Gd-IgA1 deposition.

In this study, the diagnosis of LRV entrapment was made by 2 well experienced sonologists through comprehensive judgment according to the diagnostic criteria. A total of 47 patients were diagnosed with LRV entrapment in our cohort, with the prevalence rate of 5.9%. Patients’ age ranged from 15 to 67 years old, but usually presented in slim females in their second to fourth decade of life, which were consistent with previous reports [24–26].

LRV entrapment was found to coexist with several kinds of renal diseases. Of 68% of the LRV entrapments (32/47) were combined with IgAN and HSPN, although these two types of renal disease were not the leading type of glomerulonephritis in our patients, which was probably due to the changes in the spectrum of kidney diseases in recent years in China [27,28]. Since IgAN and HSPN were considered to be different manifestations of a single disease process, we merged patients with idiopathic IgAN and HSPN into one group (expanded IgAN group) for statistical analysis. We found that the frequency of LRV entrapment in expanded IgAN group was significantly higher than that in patients with non-expanded IgAN, even after adjustment for age, gender, and BMI by PSM analysis. Our results indicated LRV entrapment was more common in expanded IgAN, and the higher prevalence was independent on the distribution characteristics of age, gender, and BMI of patients, which supported the hypothesis of the causal association between LRV entrapment and the presence of IgAN.

Subclinical mesangial IgA deposition has been observed in 4–16% of the general population *via* assessment of renal specimens obtained through necropsy of patients without any manifestation of renal disease [29,30]. There have been a few studies to explore the clinical significance of the latent glomerular IgA deposition. Koichi Suzuki’s cohort suggested the latent mesangial IgA deposition was associated with a mild degree of hematuria [31]. Latent mesangial IgA deposition in donor kidneys at transplantation has been reported to be associated with a worse outcome of allograft survival [32]. These results indicated the potential pathogenicity of incidental glomerular IgA deposition. Besides IgAN, HSPN, and LN, glomerular incidental IgA deposition was also found in several types of glomerular disease, such as PMN and ANCA-associated vasculitis [16]. In our cohort, glomerular IgA deposition was observed in 9.5% of patients with LN and IgAN-unrelated diseases, and patients combined with LRV entrapment were significantly much more often to have glomerular incidental IgA deposition.

Gd-IgA1 is suggested to play a key role in glomerular deposition of IgA1-containing immune complex and subsequent renal inflammation in IgAN and HSPN. The results of Gd-IgA1 staining in secondary IgAN and incidental IgA deposition were controversial. Suzuki et al. reported that Gd-IgA1 findings were negative in all adult patients with LN (*n* = 7), PMN (*n* = 1), and acute poststreptococcal glomerulonephritis (*n* = 1) with IgA deposition [16]. Wada et al. found renal Gd-IgA1 deposition was apparently specific to IgAN (*n* = 50) and HSPN (*n* = 18) at higher intensity, and Gd-IgA1 staining was more intense in these groups than in LN (*n* = 3) or minimal change disease (*n* = 3) [33]. Zhao et al. demonstrated that Gd-IgA1 staining intensity was observed in LN patients (*n* = 11), weak or negative in incidental IgA deposition (*n* = 13) [34]. In pediatric patients, Ishiko et al. reported Gd-IgA1 staining was positive in patients with LN (*n* = 9) and MN (*n* = 1), but negative in idiopathic nephrotic syndrome (*n* = 6) and Alport syndrome (*n* = 1) with IgA deposition [35]. Wang et al. demonstrated 3 blood-related living renal allograft donors with subclinical mesangial IgA deposition had weak glomerular Gd-IgA1 [36]. Limited sample sizes and different population might contribute to the discrepancy. In a recent published case report, Lee et al. showed three cases with glomerular Gd-IgA1 positive complicated with rheumatoid arthritis, systemic lupus erythematosus, and Crohn’s disease achieved clinical renal remission treated with tonsillectomy and steroid pulse therapy, while another case with hepatitis C showed negative for Gd-IgA1 and successfully treated by antivial agents [36]. Although these small number of cases were not enough to prove that Gd-IgA1 was a strong tool to differentiate primary IgAN from secondary IgAN, they indeed indicated the relatively high difficulty of exactly differentiating primary and secondary IgAN at present, which might another important factor for the discrepancy of Gd-IgA1 staining. Wang et al. showed renal allograft donors with latent IgA deposition had similar immune features to patients with IgAN, including increased plasma levels of IgA, IgA1, Gd-IgA1, and renal Gd-IgA1 deposition, donors with IgA deposition had lower levels of antiglycan antibodies, which may be one explanation for the subclinical status of IgA deposition in donors [37]. Whatever, glomerular Gd-IgA1 deposition might be a prerequisite step in the pathogenesis of IgAN. In the local clinical practice, Gd-IgA1 staining was not performed routinely, glomerular IgA deposition accompanied with other glomerulonephritis without obvious manifestation of IgAN, such as mesangial cell proliferation and matrix expansion, was considered as incidental IgA deposition. Interestingly, when KM55 staining was added, Gd-IgA1 was positive in all patients accompanied by LRV entrapment and glomerular incidental IgA deposition. The pathological types of the five patients were 3 PMN, 1 TIN, and 1 C3 glomerulonephritis, with no secondary IgAN. To match the glomerular disease types, we randomly chose 17 PMN patients accompanied by incidental IgA deposition to perform KM55 staining, and Gd-IgA1 was only detected in five patients. Our results demonstrated that in patients with incidental glomerular IgA deposition, those with LRV entrapment were more often to have Gd-IgA1 deposition, although we could not make sure whether these patients were indeed combined with IgAN.

The limited number of patients with LRV entrapment did not allow us to do credible comparisons of clinical and histological data between patients with and without LRV entrapment in IgAN and HSPN. It is our future task to elucidate whether LRV entrapment influences the severity and progression of the diseases.

This is a preliminary study with relatively limited number of patients with LRV entrapment, especially non-IgAN patients concurrent with LRV entrapment and glomerular incidental IgA deposition. Multicenter and large sample-sized studies with long-term follow-up would aid in the understanding of this rare entity in renal diseases.

## Conclusions

In conclusion, LRV entrapment coexisted with several kinds of renal diseases, with a significantly higher prevalence in patients with idiopathic IgAN and HSPN. In patients of LN and IgAN-unrelated disease with LRV entrapment, glomerular IgA and Gd-IgA1 deposition were more common compared with patients without LRV entrapment. The causal relationship of LRV entrapment and the development of IgAN and HSPN deserved to be further investigated.

## Supplementary Material

Supplemental Material

## Data Availability

The datasets used and analyzed during this study are available from the corresponding author on reasonable request.
