# Supplementary material for: Associations of left renal vein entrapment with IgA nephropathy and Henoch–Schönlein purpura nephritis
Source: Ren Fail. 2022 Sep 7;44(1):1519–27. doi: 10.1080/0886022X.2022.2118065 (PMC9467612; doi:10.1080/0886022X.2022.2118065)
Supplement: Supplemental Material [file IRNF_A_2118065_SM0869.pdf]

**Supplementary Table 1. Clinicopathological features of patients with and without LRV entrapment in IgAN and HSPN**

| | IgAN with LRV (n=22) | IgAN without LRV (n=130) | HSPN with LRV (n=10) | HSPN without LRV (n=25) |
|---|---|---|---|---|
| **Clinical data** Sex (female/male) | 15/7 | 55/75* | 7/3 | 11/14 |
| Age (years) | 30.1 ± 11.3 | 40.4 ± 13.5* | 29.1 ± 17.4 | 36.9 ± 18.4 |
| BMI (kg/m$^2$) | 20.2 ± 3.0 | 25.3 ± 3.8* | 19.3 ± 1.5 | 26.1 ± 3.3* |
| Hematuria per high power | 336.7 ± 608.2 | 44.0 ± 109.7 | 27.4 ± 29.8 | 34.8 ± 37.1 |
| 24-hour urine protein (g/d) | 2.1 ± 2.0 | 2.6 ± 3.1 | 0.8 ± 0.4 | 1.7 ± 1.1* |
| Albumin (g/l) | 36.4 ± 6.9 | 36.3 ± 6.6 | 42.5 ± 4.2 | 37.6 ± 5.4* |
| Serum creatinine (μmol/l) | 106.9 ± 72.2 | 103.2 ± 52.9 | 66.4 ± 26.7 | 82.4 ± 31.2 |
| eGFR (ml/min/1.73m$^2$) | 95.1 ± 45.8 | 82.9 ± 32.2 | 113.9 ± 31.2 | 98.1 ± 30.2 |
| Systolic blood pressure(mmHg) | 130.1 ± 12.7 | 132.4 ± 18.1 | 124.3 ± 21.1 | 136.3 ± 26.8 |
| Diastolic blood pressure(mmHg) | 93.3 ± 12.0 | 87.0 ± 11.5 | 79.0 ± 3.6 | 84.0 ± 11.0 |
| **Pathological data** | | | | |
| IgA deposition | 2 (1, 3) | 3 (1,4) | 2(1,3) | 2(1,3) |
| M score | 1 (0, 1) | 1 (0,1) | 0(0,1) | 0(0,1) |
| E score | 1 (0, 1) | 0 (0,1) | 0 (0,1) | 1(0,1) |
| S score | 1 (0, 1) | 1 (0,1) | 1 (0,1) | 1 (0,1) |
| T score | 0 (0, 2) | 0 (0,2) | 0 (0,1) | 1 (0,1) |
| C score | 0 (0, 1) | 0 (0,2) | 0 (0,1) | 0 (0,1) |

**Abbreviations: LRV:** left renal vein; **BMI:** body mass index; **IgAN:** IgA nephropathy; **HSPN:** Henoch-schonlein purpura nephritis; **eGFR:** estimated glomerular filtration rate; **M:** mesangial hypercellularity; **E:** endocapillary hypercellularity; **S:** segmental sclerosis; **T:** tubular atrophy/interstitial fibrosis; **C:** crescents. *$P$<0.05

Information Classification: General
